# On cycle-free accessible union stable network structures

**DOI:** 10.1007/s10288-025-00594-y

**Published:** 2025-09-08

**Authors:** Encarnación Algaba, René van den Brink, Chris Dietz

**Affiliations:** 1 Matemática Aplicada II and Instituto de Matemáticas de la Universidad de Sevilla (IMUS), Escuela Superior de Ingenieros, Camino de los Descubrimientos, s/n, 41092 Sevilla, Spain; 2 Department of Economics and Tinbergen Institute, VU University Amsterdam, De Boelelaan 1105, 1081 HV Amsterdam, the Netherlands

**Keywords:** Cooperative game, Cycle-free graph, Accessible union stable structure, Shapley value, Axiomatization, 91A12, 91A43, 05C21

## Abstract

We investigate set theoretic properties that characterize the collection of connected coalitions in cycle-free undirected graphs among the accessible union stable network structures. It turns out that the only additional requirement is that every non-unitary feasible coalition can be written in a unique way as a union of non-unitary supports. As a consequence, a fairness axiom for solutions for cooperative games on cycle-free accessible union stable network structures can be defined that, together with the well-known component efficiency, characterizes the Shapley value on the class of cycle-free accessible union stable network structures. Since this fairness axiom combines ideas behind the traditional fairness axiom and balanced contributions, we refer to it as *balanced fairness*.

## Introduction

A *cooperative game with transferable utility*, or simply a TU-game, consists of a finite set of players and for any subset (coalition) of players a worth representing the total payoff that the coalition can obtain by cooperating. A main question is how to allocate the worth that all players cooperating together can earn over the individual players. In its classical interpretation, a TU-game describes a situation in which every coalition *S* (i.e subset) of *N* can be formed and earn its worth. In the literature various restrictions on coalition formation have been developed. Two main forms of restricted cooperation that have been studied are communication restrictions and hierarchies. Myerson (1977) introduced the well-known model of a communication graph game^1^ that consists of a TU-game and an undirected (communication) graph where it is assumed that only coalitions that are connected in the communication graph are feasible. A restricted game is defined where the worth of every feasible (i.e. connected) coalition equals its worth in the original game, while the worth of a nonconnected coalition equals the sum of the worths of its maximally connected subsets (also known as components) of the coalition. Further, he showed that the solution that assigns to every *communication graph game* the Shapley value of the restricted game is the only solution that satisfies component efficiency (meaning that every maximally connected subset of players earns its own worth) and fairness (requiring that deleting a communication link between two players has the same effect on the individual payoffs of these two players).^2^ Besides, Myerson (1980) characterized this solution by component efficiency and balanced contributions (meaning that the effect of the isolation of one player on the payoff of another player, is the same as the effect of the departure of the second player on the payoff of the first player). Algaba et al. (2000, 2001) generalize this model to games on *union stable network structures*^3^ being network structures that satisfy the property that the union of every pair of nondisjoint feasible coalitions is also feasible. They also generalize the above mentioned characterizations of the Shapley value for such games, and with the support basis establish the connection with hypergraphs in Algaba et al. (2004).^4^ Later, Algaba et al. (2015) generalize the Shapley value by introducing the Harsanyi power solutions in this context^5^, extending the work on communication graphs by van den Brink et al. (2011).

A model that studies restrictions in cooperation arising from hierarchies is that of a *game with a permission structure*. In those games it is assumed that the players are part of a hierarchical organization, where some players need permission or approval from other players before they are allowed to cooperate. In the conjunctive approach as developed in Gilles et al. (1992) and van den Brink and Gilles (1996), it is assumed that each player needs permission from *all*its predecessors before it is allowed to cooperate with other players. This implies that a coalition is feasible if and only if for every player in the coalition it holds that all its predecessors belong to the coalition. Alternatively, in the disjunctive approach as developed in Gilles and Owen (1994) and van den Brink (1997), see also Gilles (2010), it is assumed that each player (except the top-players) needs permission from *at least one*of its predecessors before it is allowed to cooperate. Consequently, a coalition is feasible if and only if every (non-top) player in the coalition has at least one predecessor who also belongs to the coalition. In Algaba et al. (2004) it is shown that the sets of feasible coalitions arising from these permission structures are *antimatroids* being well-known combinatorial structures representing hierarchies, see Dilworth (1940) and Edelman and Jamison (1985). A set of feasible coalitions is an antimatroid if it contains the empty set, satisfies *accessibility* (meaning that every nonempty feasible coalition has at least one player that can leave the coalition and the result is a feasible subcoalition) and is *union closed* (establishing that the union of two feasible coalitions is also feasible). An overview of games on hierarchies is given in van den Brink (2017)^6^. Antimatroids satisfying union closedness directly shows that these structures, and thus also permission structures, are a special class of union stable network structures.

In the field of restricted cooperation, van den Brink (2012) made clear the distinction between hierarchies and communication networks by showing that the network structures that can be the set of connected coalitions in some undirected graph are exactly those network structures that, besides containing the empty set, satisfy the above mentioned union stability and *2-accessibility* (requiring that every feasible coalition with two or more players has at least two players that can leave the coalition such that the remaining set of players is still a feasible coalition). So, compared to communication feasible sets (network structures that can be obtained as the set of connected coalitions in some undirected graph), antimatroids satisfy a stronger union property (since union closedness implies union stability) but a weaker accessibility property (since two-accessibility implies accessibility).

In view of this last result, Algaba et al. (2018) considered the so-called *accessible union stable network structures* being network structures that take the weaker of the two union and accessibility properties for network structures considered above, i.e. union stability (from communication feasible sets) and accessibility (from antimatroids)^7^. They also characterized a Shapley value for these games using a balanced contributions axiom.^8^ This is possible since after isolating a player in an accessible union stable network structure, what is left is still an accessible union stable network structure. As mentioned in Algaba et al. (2018), this is not the case when we delete all feasible coalitions that contain two specific players, i.e. the resulting structure need not be an accessible union stable network structure.

Although properties as accessibility and union stability reflect two real common and desirable features (hierarchy and communication, respectively) in the design and analysis of real networks, when generating new networks from existing ones, these might not keep both properties. This brings complications when one has to stay within the class, for example when axiomatizing an allocation rule. However, in this work, we find a framework where these two specific properties are preserved, proving that the cycle-freeness property plays a remarkable role to maintain these two properties. In fact, we characterize the property of cycle freeness for accessible union stable networks in comparison with the cycle freeness property for the larger class of union stable networks as well as the smaller class of those networks derived from a communication graph. More concretely, it turns out that, with accessibility and union stability, the additional cycle-freeness (i) implies the two-intersection closedness property that Algaba et al. (2001) had to additionally require to extend cycle-free communication graphs within the class of union stable network structures, and (ii) implies that the network structure can be obtained as the set of connected coalitions in a cycle-free undirected graph. Additionally, we show that accessibility and union stability are inherited when deleting all coalitions containing two specific players if these two players form a support and the structure is cycle-free, in the sense that every non-unitary feasible coalition can be written in a unique way as a union of non-unitary supports. As a consequence, a fairness axiom for solutions for cooperative games on cycle-free accessible union stable network structures can be defined that says that deleting all feasible coalitions containing two specific players has the same effect on the payoffs of these two players. Since this fairness axiom combines ideas behind the traditional fairness axiom and balanced contributions, we refer to it as *balanced fairness*. Together with the well-known component efficiency and component dummy, this axiom characterizes the Shapley value on the class of cycle-free accessible union stable network structures.

This note is organized as follows. Section 2 contains preliminaries on cooperative games, network structures and allocation rules for games with restricted cooperation. In Sect. 3, we discuss structural properties of cycle-free accessible union stable network structures, while in Sect. 4, we discuss the impact on axiomatizations of the Shapley value for such games. Finally, Sect. 5 contains concluding remarks.

## Preliminaries

### Cooperative games

A situation in which a finite set of players can obtain certain payoffs by cooperation can be described by a *cooperative game with transferable utility*, or simply a TU-game, being a pair (*N*, *v*), where $N \subseteq \mathrm{I\hspace{-2.29996pt}N}$ is a finite set of players and $v :2^N \rightarrow \mathrm{I\hspace{-2.20001pt}R}$ is a *characteristic function* on *N* satisfying $v(\emptyset ) = 0$. For any *coalition* $S \subseteq N$, *v*(*S*) is the *worth* of coalition *S*, meaning that the members of coalition *S* can obtain a total payoff of *v*(*S*) by agreeing to cooperate. Since we take the player set to be fixed, we denote a TU-game (*N*, *v*) just by its characteristic function *v*. We denote the collection of all TU-games on player set *N* by ${{\mathcal {G}}}^N$.

A *payoff vector* of an *n*-player TU-game $v \in {{\mathcal {G}}}^N$ is an *n*-dimensional vector $x \in \mathrm{I\hspace{-2.20001pt}R}^N$ giving a payoff $x_i \in \mathrm{I\hspace{-2.20001pt}R}$ to any player $i \in N$. A (single-valued) *solution* for TU-games is a mapping *f* that assigns to every game $v \in {{\mathcal {G}}}^N$ a payoff vector $f(v) \in \mathrm{I\hspace{-2.20001pt}R}^N$. One of the most well-known and important solution for TU-games is the *Shapley value* (Shapley 1953) given, for all $i \in N$, by

1 $$\begin{aligned} Sh_i(v) = \sum _{{S \subseteq N,}{i \in S}} \frac{( |N| - |S| )!( |S| -1)!}{ |N| !} (v(S) - v(S \setminus \{i\})). \end{aligned}$$

In Algaba et al. (2020), the versatility and popularity of this value is highlighted with a wide collection of theoretical and applied results.

### Network structures

In a TU-game any subset $S \subseteq N$ is assumed to be able to form a coalition and earn the worth *v*(*S*). However, in most economic and political organizations not every set of participants can form a feasible coalition. For a finite set *N*, a *network structure* over *N* is a pair $\left( N,{{\mathcal {F}}}\right)$ where ${{\mathcal {F}}} \subseteq 2^{N}$ is a family of subsets. The sets belonging to ${{\mathcal {F}}}$ are called *feasible* coalitions. A network structure $\left( N,{{\mathcal {F}}}\right)$ is called normal if $N=\bigcup \nolimits _{S\in {{\mathcal {F}}}}S,$ i.e. if every player belongs to at least one feasible coalition.

A triple $(N,v,{{\mathcal {F}}})$ with $v \in {{\mathcal {G}}}^N$ and ${{\mathcal {F}}} \subseteq 2^N$ is called a *game with restricted cooperation*. Again, since we take the player set to be fixed, we denote a game with restricted cooperation $(N,v,{{\mathcal {F}}})$ by $(v,{{\mathcal {F}}})$.

One of the most well-known models of restricted cooperation are the games with communication restrictions as introduced in Myerson (1977). In this model a *communication network*, represented by an undirected graph, on the set of players in a cooperative game is given, and a coalition *S* is feasible if and only if the players in *S* are connected within this communication network.

An *undirected graph* is a pair (*N*, *L*) where *N* is the set of nodes and $L \subseteq \{\{i,j\}|i,j \in N,~i \ne j\}$ is a collection of subsets of *N* such that each element of *L*,  called *edge* or *link*, contains precisely two nodes. Since in this paper the nodes in a graph represent the positions of players in a network we refer to them as players. A sequence of *k* different players $(i_1,\ldots ,i_k)$ is a *path* in (*N*, *L*) if $\{i_h,i_{h+1}\} \in L$ for $h=1,\ldots ,k-1$. Two distinct players *i* and *j*, $i \ne j$, are *connected* in graph (*N*, *L*) if there is a path $(i_1,\ldots ,i_k)$ with $i_1=i$ and $i_k=j$. A coalition $S \subseteq N$ is *connected* in graph (*N*, *L*) if every pair of players in *S* is connected by a path that only contains players from *S*, i.e. for every $i,j \in S,\ i \ne j$, there is a path $(i_1,\ldots ,i_k)$ such that $i_1=i,\ i_k=j$ and $\{i_1,\ldots ,i_k\} \subseteq S$. A maximally connected subset *T* of coalition *S* in (*N*, *L*) is called a *component* of *S* in that graph, i.e., $T \subseteq S$ is a component of *S* in (*N*, *L*) if and only if (i) *T* is connected in (*N*, *L*(*S*)) and (ii) for every $h \in S \setminus T$ the coalition $T \cup \{h\}$ is not connected in (*N*, *L*(*S*)), where $L(S) = \{\{i,j\} \in L| \{i,j\} \subseteq S\}$ is the set of links between players in *S*.

A sequence of players $(i_1,\ldots ,i_{k},i_1)$, $k \ge 2$, is a *cycle* in (*N*, *L*) if $(i_1,\ldots ,i_k)$ is a path in (*N*, *L*) and $\{i_{k},i_1\} \in L$. A graph (*N*, *L*) is *cycle-free* when it does not contain any cycle.

A triple (*N*, *v*, *L*) with (*N*, *v*) a TU-game and (*N*, *L*) an undirected communication graph is called a *communication graph game*. Since, again we take the player set to be fixed, we denote a communication graph game (*N*, *v*, *L*) just by (*v*, *L*). In the communication graph game (*v*, *L*) players can cooperate if and only if they are able to communicate with each other. Hence, the set of feasible coalitions in (*N*, *v*, *L*) is given by

$$\begin{aligned} {{\mathcal {F}}}_L = \{S \subseteq N \mid S \text{ is } \text{ connected } \text{ in } (N,L)\}. \end{aligned}$$

We refer to this set as the *communication feasible set* of communication graph (*N*, *L*). Myerson (1977) introduced the *restricted game* of a communication graph game (*v*, *L*) as the TU-game $$v_L$$ in which every feasible coalition *S* can earn its worth *v*(*S*). Whenever *S* is not feasible it can earn the sum of the worths of its components in (*N*, *L*) . Denoting the set of components of $$S \subseteq N$$ in (*N*, *L*) by $$ C_L(S)$$, the restricted game $$v_L$$ is given by^9^

2 $$\begin{aligned} v_{L}(S)=\sum _{T\in C_{L}(S)}v(T) \text{ for } \text{ all } S \subseteq N. \end{aligned}$$

The solution $\mu$ given by Myerson (1977) is obtained by taking for every communication graph game, the Shapley value of the corresponding restricted game, later called the *Myerson value* for communication graph games, i.e., $\mu (v,L)=S\!h(v_L)$.

Hierarchies can be modeled by *antimatroids* that were introduced by Dilworth (1940) as particular examples of semimodular lattices. Since then, several authors have obtained the same concept by abstracting various combinatorial situations (see Korte et al. 1991 and Edelman and Jamison 1985).

#### Definition 2.1

A network structure ${{\mathcal {A}}} \subseteq 2^N$ is an antimatroid if it satisfies

(i) (feasible empty set) $\emptyset \in {{\mathcal {A}}}$,
(ii) (union closedness) $S,T\in {{\mathcal {A}}}$ implies that $S\cup T\in {{\mathcal {A}}},$ and
(iii) (accessibility) $S\in {{\mathcal {A}}}$ with $S\ne \emptyset$, implies that there exists $i\in S$ such that $S\setminus \{i\}\in {{\mathcal {A}}}$.

Algaba et al. (2004) consider a model where cooperation in a cooperative game is restricted by the players being members of a hierarchical structure represented by an antimatroid. These *games on an antimatroid* generalize the model of a game with an acyclic permission structure, see Gilles et al. (1992) and Gilles and van den Brink (1996) for the conjunctive approach and Gilles and Owen (1994) and van den Brink (1997) for the disjunctive approach

Algaba et al. (2000, 2001) introduce *games on a union stable network structure* which generalize both the communication graph games as well as games on antimatroids (and hence, games with a permission structure), establishing its relationship with hypergraphs in Algaba et al. (2004).

#### Definition 2.2

A network structure ${{\mathcal {F}}} \subseteq 2^N$ is *union stable* if it satisfies

(i) (feasible empty set) $\emptyset \in {{\mathcal {F}}}$,
(ii) (union stability) $S,T\in {{\mathcal {F}}}$ with $S \cap T \ne \emptyset$, implies that $S\cup T\in {{\mathcal {F}}}$.

In van den Brink (2012), communication feasible sets are characterized as follows.

#### Theorem 2.1

(van den Brink 2012) Let ${{\mathcal {F}}}\subseteq 2^{N}$ be a network structure on $N \subset \mathrm{I\hspace{-2.29996pt}N}$. Then ${{\mathcal {F}}}$ is the communication feasible set of some communication graph if and only if it satisfies the following properties:

(i) (feasible empty set) $\emptyset \in {{\mathcal {F}}}$,
(ii) (union stability) $S,T\in {{\mathcal {F}}}$ with $S\cap T\ne \emptyset$ implies that $S\cup T\in {{\mathcal {F}}},$
(iii) (two-accessibility) $S\in {{\mathcal {F}}}$ with $|S|\ge 2$ implies that there exist $i,j\in S$, $i\ne j$, such that $S\setminus \{ i\} \in {{\mathcal {F}}}$ and $S\setminus \{j\} \in {{\mathcal {F}}}$, and
(iv) (normality) For every $i \in N$ there is an $S \in {{\mathcal {F}}}$ such that $i \in S$.

Algaba et al. (2018) introduce the so-called accessible union stable network structures, being those network structures that satisfy the weaker union and accessibility properties when comparing properties of communication feasible sets and antimatroids.

#### Definition 2.3

A network structure ${{\mathcal {F}}} \subseteq 2^N$ is an *accessible union stable network structure* if it satisfies

(i) (feasible empty set) $\emptyset \in {{\mathcal {F}}}$,
(ii) (union stability) $S,T\in {{\mathcal {F}}}$ with $S\cap T\ne \emptyset ,$ implies that $S\cup T\in {{\mathcal {F}}},$ and
(iii) (accessibility) $S\in {{\mathcal {F}}}$ with $S\ne \emptyset$, implies that there exists $i\in S$ such that $S\setminus \{i\}\in {{\mathcal {F}}}$.

Obviously, antimatroids and communication feasible sets are accessible union stable network structures.

### Axioms for allocation rules

Myerson (1977) characterized the Myerson value for communication graph games by component efficiency (meaning that the sum of the payoffs of the players in any graph component equals the worth of that component in the original game) and fairness (meaning that deleting a link from the undirected communication graph has the same effect on the payoffs of the two players connected by that link).

Similar as the set of edges of an undirected graph determine the set of all connected coalitions, a union stable network structure can be fully determined by its *basis* as in Algaba et al. (2000, 2001). For each union stable network structure ${{\mathcal {F}}}$, the set

$$\begin{aligned} {{\mathcal {E}}}\left( {{\mathcal {F}}}\right) =\{G\in {{\mathcal {F}}}:G=A\cup B,\;A\ne G,\;B\ne G,\;A,B\in {{\mathcal {F}}},\;A\cap B\ne \emptyset \}, \end{aligned}$$

is the set of *supportable coalitions*, being those coalitions in ${{\mathcal {F}}}$ that can be written as the union of two other non disjoint feasible coalitions. The set ${{\mathcal {B}}}\left( {{\mathcal {F}}}\right) ={{\mathcal {F}}}\setminus {{\mathcal {E}}}\left( {{\mathcal {F}}}\right) ,$ is called the *basis* of ${{\mathcal {F}}}$, and the elements of ${{\mathcal {B}}}\left( {{\mathcal {F}}}\right)$ are called *supports* of ${{\mathcal {F}}}$.

As mentioned, the set of connected coalitions in an undirected graph or communication feasible set, is a union stable network structure. The basis of a communication feasible set is exactly the set of edges (feasible coalitions of size two), the singletons (feasible coalitions of size one), and the empty set. The components of an undirected graph can be generalized to union stable network structures as follows. Let ${{\mathcal {F}}}\subseteq 2^{N}$ be a network structure and let $S\subseteq N$. A set $T\subseteq S$ is called a ${{\mathcal {F}}}$-*component* of *S* if $T\in {{\mathcal {F}}}$ and there exists no $T^{\prime }\in {{\mathcal {F}}}$ such that $T\subset T^{\prime }\subseteq S$. Therefore, the ${{\mathcal {F}}}$-components of *S* are the maximal feasible coalitions that belong to ${{\mathcal {F}}}$ and are contained in *S*. We denote by $C_{{{\mathcal {F}}}}(S)$ the collection of the ${{\mathcal {F}}}$-components of *S*. Union stable network structures can be characterized in terms of the ${{\mathcal {F}}}$-components of a coalition in the following way: The network structure ${{\mathcal {F}}}\subseteq 2^{N}$ is union stable if and only if for any $S\subseteq N$ with $C_{{{\mathcal {F}}}}(S)\ne \emptyset$, the ${{\mathcal {F}}}$-components of *S* are a collection of pairwise disjoint subsets of *S*.

An *allocation rule* or value for a class of games with restricted cooperation ${{\mathcal {C}}}$ is a function $f:{{\mathcal {C}}}\rightarrow \mathrm{I\hspace{-2.20001pt}R}^{N}$ such that $f(v,{{\mathcal {F}}})\in \mathrm{I\hspace{-2.20001pt}R}^{N}$ for all $(v,{{\mathcal {F}}}) \in {{\mathcal {C}}}$, that assigns a payoff vector to every game in this class. Algaba et al. (2001) used the components to extend the definition of the Myerson value to games on union stable network structures by applying the Shapley value to the restricted game $v^{{{\mathcal {F}}}}:2^{N}\rightarrow \mathrm{I\hspace{-2.20001pt}R},$ defined by

3 $$\begin{aligned} v^{{{\mathcal {F}}}}(S)=\sum _{T\in C_{{{\mathcal {F}}}}\left( S\right) }v(T)\;\; \text{ for } \text{ all } S\subseteq N. \end{aligned}$$

They also extend the axiomatization of the Myerson value by component efficiency (meaning that in every component exactly the worth of that component is allocated over the players in the component), component dummy (requiring that a player who does not belong to any feasible coalition earns a zero payoff^10^), and fairness (establishing that deleting one support from the basis of ${{\mathcal {F}}}$ and taking the union stable closure^11^ of the new basis, the payoffs of all players in the support that is deleted change by the same amount).

## Cycle-free accessible union stable network structures

To generalize the cycle-free undirected graphs within the class of union stable network structures, Algaba et al. (2000) introduced the subclass of union stable network structures that are (i) intersection closed if the intersection contains at least two elements and (ii) such that every non-unitary feasible coalition can be written in a unique way as a union of non-unitary supports. A nonempty coalition *S* is non-unitary if $|S| \ne 1$ (i.e. *S* is not a singleton).

### Definition 3.1

A union stable network structure ${{\mathcal {F}}} \subseteq 2^N$ belongs to the subclass of union stable network structures $USI^N$ if the following two conditions are satisfied^12^:

(i) (two-intersection closed) $S,T\in {{\mathcal {F}}}$ with $\left| S\cap T \right| \ge 2$ implies that $S\cap T\in {{\mathcal {F}}}$,
(ii) (cycle-free) every non-unitary feasible coalition can be written in a unique way as a union of non-unitary supports.

Note that for network structures ${{\mathcal {F}}}$ in this class, if $S, T \in B({{\mathcal {F}}})$, $|S| \ge 2$, then $S \not \subset T$ (since otherwise *T* could be written as $T \cup \emptyset$ as well as $T \cup S$, and then the representation would not be unique.) This subclass of union stable network structures contains the important class of connected coalitions in a cycle-free communication graph, see e.g., Le Breton et al. (1992) and Demange (1994, 2004).

We remark that Algaba et al. (2000) did not give names to the two network properties in Definition 3.1. Our motivation to refer to this second property as cycle-freeness comes from the interesting observation that adding this property to those that characterize communication feasible sets in Theorem 2.1, characterizes the sets of coalitions that can be the set of connected coalitions in a cycle-free graph.

### Proposition 3.1

Let ${{\mathcal {F}}} \subseteq 2^N$ be a communication feasible set, i.e. there is an undirected graph *L* such that ${{\mathcal {F}}}={{\mathcal {F}}}_L$. Then *L* is cycle-free if and only if every non-unitary feasible coalition in ${{\mathcal {F}}}$ can be written in a unique way as a union of non-unitary supports (i.e., ${{\mathcal {F}}}$ is cycle-free).

### Proof

Let ${{\mathcal {F}}} \subseteq 2^N$ be such that there is an undirected graph *L* with ${{\mathcal {F}}}={{\mathcal {F}}}_L$. **(Only if)** Suppose that *L* is cycle-free. Then for every connected coalition $S \in {{\mathcal {F}}}$ the unique way to write *S* as union of non-unitary supports is $S=\bigcup _{l \in L(S)} l$.

**(If)** Suppose that *L* is not cycle-free. Let $(i_1, i_2, \ldots , i_k, i_1),\ k \ge 3$, be a cycle in *L*. Then $S=\bigcup _{t \in \{1, \ldots , k-1\}} \{i_t,i_{t+1}\} = \{i_k,i_1\} \cup \left( \bigcup _{t \in \{2, \ldots , k-1\}} \{i_t,i_{t+1}\}\right)$, and thus *S* can be written as a union of non-unitary supports in at least two ways. $\square$

In van den Brink (2012) it is shown that adding intersection closedness to the properties which characterize a communication feasible set, characterizes the sets of coalitions that can be the set of connected coalitions in a cycle-complete graph. A graph is *cycle-complete* if, whenever there is a cycle, the subgraph on that cycle is complete.^13^

### Proposition 3.2

(van den Brink 2012) Let ${{\mathcal {F}}} \subseteq 2^N$ be a communication feasible set, i.e. there is an undirected graph *L* such that ${{\mathcal {F}}}={{\mathcal {F}}}_L$. Then there is a cycle-complete graph *L* such that ${{\mathcal {F}}}={{\mathcal {F}}}_L$ if and only if ${{\mathcal {F}}}$ is intersection closed.

Since for communication feasible sets, all singletons are feasible, Proposition 3.2 also holds if we use the weaker two-intersection closedness instead of intersection closedness.^14^ Since every cycle-free graph is cycle-complete, from Propositions 3.1 and 3.2, it follows that, under the properties that characterize a communication feasible set, cycle-freeness of ${{\mathcal {F}}} \subseteq 2^N$ implies intersection closedness of ${{\mathcal {F}}}$ but not the other way around. For example, ${{\mathcal {F}}} = 2^N$ is intersection closed, but not cycle-free. So, when we want to consider a generalization of cycle-free undirected graphs (as the class $USI^N$) within the class of accessible union stable network structures, two-intersection closedness is superfluous. However, it is important to emphazise that for arbitrary union stable network structures both properties are independent, i.e., cycle-freeness does not imply two-intersection closedness (and thus not intersection closedness), as shown in the following example.

### Example 3.1

Consider a market with one buyer (player 1), two sellers (players 2 and 3) and one intermediary (player 4). Suppose that a nonempty coalition $S \subseteq 2^N,\ N=\{1,2,3,4\}$, is feasible if and only if it contains the buyer, the intermediary and at least one of the two sellers, i.e.,

$$\begin{aligned} {{\mathcal {F}}}=\{\emptyset , \{1,2,4\},\{1,3,4\},\{1,2,3,4\}\}, \end{aligned}$$

with basis $B({{\mathcal {F}}})=\{\emptyset , \{1,2,4\},\{1,3,4\}\}$. This is a union stable network structure that is cycle-free. However, note that it is not two-intersection closed (since $\{1,2,4\} \cap \{1,3,4\} = \{1,4\}$ which is not feasible). $\square$

Algaba et al. (2018) showed that the supports of an accessible union stable network structure are either of cardinality at most equal to 2 or *paths*, i.e., coalitions containing a unique player that can leave the coalition keeping feasibility.^15^ The next proposition investigates how the property of cycle-freeness affects an accessible union stable network, in the sense that for accessible union stable network structures, cycle-freeness implies that every nonempty support contains at most two players.

### Proposition 3.3

Let ${{\mathcal {F}}} \subseteq 2^N$ be an accessible union stable network structure that is cycle-free (i.e. all non-unitary feasible coalitions can be written in a unique way as a union of non-unitary supports). If $S \in B({{\mathcal {F}}})$ then $|S| \le 2$.

### Proof

Let ${{\mathcal {F}}} \subseteq 2^N$ be an accessible union stable network structure that is cycle-free. Suppose there is an $S \in B({{\mathcal {F}}})$ such that $|S|>2$. By accessibility of ${{\mathcal {F}}}$ there is a $T \in {{\mathcal {F}}}$ such that $T=S \setminus \{i\}$ for some $i \in S$. By cycle-freeness and $T \subset S$, it holds that $T \not \in B({{\mathcal {F}}})$. But then, there exist nondisjoint supports $T_1, T_2, \ldots , T_k \in B({{\mathcal {F}}})$, $k \ge 2$ with $T_l \cap T_p \ne \emptyset$ for all $l, p \in \{1, \ldots , k\}$, such that $T=\bigcup _{p=1}^k T_p$. But then, the supports $T_1, \ldots , T_k$ are subsets of *S* which contradicts cycle-freeness of ${{\mathcal {F}}}$. $\square$

Proposition 3.3 implies that a cycle-free accessible union stable network structure in which all singletons are feasible is the set of connected coalitions in some cycle-free graph. Since, as mentioned in the paragraph after Proposition 3.2, under the properties that characterize a communication feasible set, cycle-freeness of ${{\mathcal {F}}} \subseteq 2^N$ implies intersection closedness of ${{\mathcal {F}}}$ but not the other way around, this implies that for accessible union stable network structures with all singletons feasible, cycle-freeness implies intersection closedness. Without the requirement that all singletons are feasible, the network structure is still two-intersection closed.

### Corollary 3.1

(i) Network structure ${{\mathcal {F}}} \subseteq 2^N$ is a cycle-free accessible union stable network structure such that $\{i\} \in {{\mathcal {F}}},$ for all $i \in N$ if and only if there is a cycle-free graph *L* such that ${{\mathcal {F}}}={{\mathcal {F}}}_L$.
(ii) If network structure ${{\mathcal {F}}} \subseteq 2^N$ is a cycle-free accessible union stable network structure such that $\{i\} \in {{\mathcal {F}}},$ for all $i \in N$ then ${{\mathcal {F}}}$ is intersection closed (and thus, two-intersection closed).
(iii) A network structure ${{\mathcal {F}}} \subseteq 2^N$ is a cycle-free accessible union stable network structure if and only if ${{\mathcal {F}}}$ is an accessible union stable network structure in $USI^N$.

It is interesting to point out that, in general, a cycle-free accessible union stable network structure does not necessarily satisfy intersection closedness as illustrated by the following example.

### Example 3.2

The network structure ${{\mathcal {F}}}=\{\emptyset ,\{1\},\{3\},\{1,2\},\{2,3\},\{1,2,3\}\}$ (with basis ${{\mathcal {B}}}({{\mathcal {F}}})={{\mathcal {F}}} \setminus \{\{1,2,3\}\}=\{\emptyset ,\{1\},\{3\},\{1,2\},\{2,3\}\}$) is a cycle-free accessible union stable network structure but it is not intersection closed since $\{1,2\}\cap \{2,3\}=\{2\}\notin {{\mathcal {F}}}$. $\square$

The set of connected coalitions in an undirected graph, that is not cycle-free, shows that an accessible union stable network structure with all singletons feasible has not to be cycle-free.

### Remark 3.1

Bilbao (2003) considers games on *augmenting systems*, where an augmenting system is a network structure ${{\mathcal {F}}}$ that (i) contains the empty set, (ii) is union stable, and (iii) is such that if $S, T \in {{\mathcal {F}}}$ with $S \subset T, S \ne T$, then there is an $i \in T \setminus S$ such that $S \cup \{i\} \in {{\mathcal {F}}}$. It is known from Algaba et al. (2010) that within the class of augmenting systems, it holds that $\{i\} \in {{\mathcal {F}}},$ for all $i \in N$ if and only if there is an undirected graph *L* such that ${{\mathcal {F}}}={{\mathcal {F}}}_L$. It then follows with Theorem 2.1 that a normal network structure ${{\mathcal {F}}}$ is an augmenting system with $\{i\} \in {{\mathcal {F}}}$ for all $i \in N$ if and only if ${{\mathcal {F}}}$ is a two-accessible union stable network structure. Moreover, from Proposition 3.1 and Corollary 3.1, it follows that network structure ${{\mathcal {F}}} \subseteq 2^N$ is a cycle-free augmenting system with $\{i\} \in {{\mathcal {F}}},$ for all $i \in N$ if and only if ${{\mathcal {F}}}={{\mathcal {F}}}_L,$ for some cycle-free graph *L*. Since (iii) implies the property of accessibility, we have that for augmenting systems, cycle-freeness implies two-intersection closedness.

## Cooperative games on cycle-free accessible union stable network structures

Algaba et al. (2018) also consider cooperative games restricted by accessible union stable network structures, applying the restricted game given by (3). A *game on an accessible union stable network structure* is a triple $\left( N,v, {{\mathcal {F}}}\right)$ where $v:2^{N}\rightarrow \mathrm{I\hspace{-2.20001pt}R}$ with $v(\emptyset )=0$ is the characteristic function of a cooperative TU-game, and ${{\mathcal {F}}}\subseteq 2^N$ is an accessible union stable network structure. Since, again we take the player set to be fixed, we denote a game on an accessible union stable network structure $(N,v,{{\mathcal {F}}})$ just by $(v,{{\mathcal {F}}})$. The set of all games on an accessible union stable network structure with player set *N* is denoted by $G\!AU\!S^{N}$.

We consider the allocation rule that assigns to every game on an accessible union stable network structure the Shapley value of the corresponding restricted game.

### Definition 4.1

The value $\varphi :G\!AU\!S^N\rightarrow \mathrm{I\hspace{-2.20001pt}R}^{N}$ is the allocation rule for games on accessible union stable network structures defined by $\varphi \left( v,{{\mathcal {F}}}\right) =S\!h(v^{{{\mathcal {F}}}}),$ for every $(v,{{\mathcal {F}}})\in G\!AU\!S^N$, where $S\!h$ denotes the Shapley value given by (1).

For games on an accessible union stable network structure, the value $\varphi$ generalizes the Myerson value for games restricted by communication graphs and the (conjunctive and disjunctive) permission value for games with a permission structure.

As mentioned in Sect. 2, if ${{\mathcal {F}}}$ is a union stable network structure then the components of *N* in ${{\mathcal {F}}}$ form a partition of a subset of *N*. If ${{\mathcal {F}}}$ is, moreover, normal then the components form a partition of *N*. *Component efficiency* of an allocation rule on a class of games on union stable network structures states that for every game with restricted cooperation in this class, the total payoff to every component equals its worth. We denote by $U\!S^N$ the class of all union stable network structures on *N*, and by $G\!U\!S^N$ the class of all games on union stable network structures on *N*.

### Definition 4.2

Let ${{\mathcal {C}}}\subseteq {G\!U\!S}^N$ be a class of games on union stable network structures. An allocation rule *f* on ${{\mathcal {C}}}$ satisfies component efficiency if $\sum _{i\in M}f_{i}\left( v,{{\mathcal {F}}}\right) =v(M),$ for all $\left( v,{{\mathcal {F}}}\right) \in {{\mathcal {C}}}$ and $M\in C_{{{\mathcal {F}}} }\left( N\right) .$

A player $i\in N$ is called *component dummy* in network structure ${{\mathcal {F}}}$ if this player does not belong to any feasible coalition in ${{\mathcal {F}}}$, i.e., if $i\notin \bigcup \left\{ M\in {{\mathcal {F}}}:M\in C_{{{\mathcal {F}}} }\left( N\right) \right\}$.

Note that a component dummy player in ${{\mathcal {F}}}$ is a null player in any $v^{{\mathcal {F}}}$ that is derived from some $v \in {{\mathcal {G}}}^N$.

### Definition 4.3

Let ${{\mathcal {C}}}\subseteq {GU\!S}^N$ be a class of games on union stable network structures. An allocation rule *f* on ${{\mathcal {C}}}$ satisfies component dummy if for any component dummy *i* in ${{\mathcal {F}}},$ we have $f _{i}\left( v,{{\mathcal {F}}}\right) =0$, for all $\left( v,{{\mathcal {F}}}\right) \in {{\mathcal {C}}}$.

Algaba et al. (2001) show that the Shapley value satisfies component efficiency and component dummy on $G\!U\!S^N$, and, therefore, on every ${{\mathcal {C}}}\subseteq G\!U\!S^N$. Algaba et al. (2018) provide an axiomatic characterization of the Shapley value for games on the class of all accessible union stable network structures that uses a balanced contribution axiom. Inspired by Myerson (1980), they compare the effect of isolating players in the accessible union stable network structure on each others payoff. Given a network structure ${{\mathcal {F}}} \subseteq 2^N$ and a player $i\in N,$ the network structure

$$\begin{aligned} {{\mathcal {F}}}_{-i}=\left\{ S\in {{\mathcal {F}}}\mid i\notin S\right\} , \end{aligned}$$

is given by all those feasible coalitions in ${{\mathcal {F}}}$ which do not contain player *i*.

Observe that, if ${{\mathcal {F}}} \subseteq 2^N$ is an accessible union stable network structure and $i\in N,$ then *i* is a component dummy for the accessible union stable network structure ${{\mathcal {F}}}_{-i}$ .

An allocation rule *f* on $G\!AU\!S^N$ satisfies *balanced contributions* if for every $\left( v,{{\mathcal {F}}}\right) \in G\!AU\!S^N$ and any two players $i,j\in N$ with $i\ne j,$ we have

$$\begin{aligned} f _{i}\left( v,{{\mathcal {F}}}\right) -f _{i}\left( v,{{\mathcal {F}}}_{-j}\right) = f _{j}\left( v,{{\mathcal {F}}}\right) -f _{j}\left( v,{{\mathcal {F}}}_{-i}\right) . \end{aligned}$$

Algaba et al. (2018) show the following.

### Proposition 4.1

The Shapley value is the unique allocation rule on the class of games on an accessible union stable network structure that satisfies component efficiency, component dummy, and balanced contributions.

Instead of balanced contributions, which considers the effects of deleting all coalitions containing a particular player from the set of feasible coalitions on the payoffs of another player, in this paper, we want to consider a version of fairness where we delete all coalitions containing two particular players, and require the payoffs of these two players to change by the same amount. So, for an accessible union stable network structure ${{\mathcal {F}}}$ and two players $i, j \in N$, we consider the network structure

$$\begin{aligned} {{\mathcal {F}}}_{-ij}=\{S \in {{\mathcal {F}}} \mid \{i,j\} \not \subseteq S\}, \end{aligned}$$

being the collection of feasible coalitions in ${{\mathcal {F}}}$ that do not contain both players *i* and *j*.

### Definition 4.4

Let ${{\mathcal {C}}} \subseteq GUS^N$ be a class of games on union stable network structures. An allocation rule *f* on ${{\mathcal {C}}}$ satisfies balanced fairness on ${{\mathcal {C}}}$ if

$$\begin{aligned} f_i(v,{{\mathcal {F}}}) - f_i(v,{{\mathcal {F}}}_{-ij}) = f_j(v,{{\mathcal {F}}}) - f_j(v,{{\mathcal {F}}}_{-ij}), \end{aligned}$$

for all $\left( v,{{\mathcal {F}}}\right) \in {{\mathcal {C}}}$ and $i, j \in N$ such that $\left( v,{{\mathcal {F}}}_{-ij}\right) \in {{\mathcal {C}}}$.

The restriction that $\left( v,{{\mathcal {F}}}_{-ij}\right) \in {{\mathcal {C}}}$ implies that not all feasible coalitions can be deleted.^16^ It turns out that ${{\mathcal {F}}}$ being accessible implies that ${{\mathcal {F}}}_{-ij}$ is accessible.

### Proposition 4.2

If ${{\mathcal {F}}} \subseteq 2^N$ is an accessible network structure then ${{\mathcal {F}}}_{-ij}$ is accessible.

### Proof

Let ${{\mathcal {F}}} \subseteq 2^N$ be an accessible network structure. Then $S \in {{\mathcal {F}}}_{-ij}$ implies that $S \in {{\mathcal {F}}}$, and thus there is an $h \in S$ such that $S \setminus \{h\} \in {{\mathcal {F}}}$. Since $\{i,j\} \not \subseteq S \in {{\mathcal {F}}}_{-ij}$, it holds that $S \setminus \{h\} \in {{\mathcal {F}}}_{-ij}$. So, ${{\mathcal {F}}}_{-ij}$ is accessible. $\square$

However, for an arbitrary accessible union stable network structure ${{\mathcal {F}}}$ the network structure ${{\mathcal {F}}}_{-ij}$ need not be union stable as the following example shows.

### Example 4.1

Consider the set of connected coalitions in communication graph (*N*, *L*) with $N=\{1,2,3,4\}$ and $L=\{\{1,2\},\{2,3\},\{3,4\},\{1,4\}\}$:

$$\begin{aligned} {{\mathcal {F}}}_L= &  \{\emptyset , \{1\}, \{2\}, \{3\}, \{4\}, \{1,2\}, \{1,4\}, \{2,3\}, \{3,4\},\\ &  \{1,2,3\}, \{1,2,4\}, \{1,3,4\}, \{2,3,4\}, \{1,2,3,4\}\}. \end{aligned}$$

Then ${{\mathcal {F}}}_{-12} = \{\emptyset , \{1\}, \{2\}, \{3\}, \{4\}, \{1,4\}, \{2,3\}, \{3,4\}, \{1,3,4\}, \{2,3,4\}\}$, which is not union stable since $\{1,3,4\}$ and $\{2,3,4\}$ both belong to ${{\mathcal {F}}}_{-12}$ but their union $\{1,2,3,4\}$ does not. $\square$

It turns out that union stability of the network structure ${{\mathcal {F}}}_{-ij}$ is kept if the accessible union stable network structure is cycle-free and players *i* and *j* belong to a support together.

### Proposition 4.3

If ${{\mathcal {F}}}$ is a cycle-free accessible union stable network structure, and $\{i,j\}\in B({{\mathcal {F}}})$, then ${{\mathcal {F}}}_{-ij}$ is union stable.

### Proof

Let ${{\mathcal {F}}}$ be a cycle-free accessible union stable network structure with $\{i,j\}\in B({{\mathcal {F}}})$. Take $S, T \in {{\mathcal {F}}}_{-ij}$ with $S \cap T \ne \emptyset$. We have to prove that $S \cup T \in {{\mathcal {F}}}_{-ij}$. We distinguish the following two cases.

Case 1: Suppose that $\{i,j\} \not \subseteq S \cup T$. Since $S \cup T \in {{\mathcal {F}}}$ (by union stability of ${{\mathcal {F}}}$) it holds that $S \cup T \in {{\mathcal {F}}}_{-ij}$.

Case 2: Suppose that $\{i,j\}\subseteq S\cup T$. Since $S,T\in {{\mathcal {F}}}_{-ij}$ it holds that $|S\cap \{i,j\}|=|T\cap \{i,j\}|=1$ with $S\cap \{i,j\}\ne T\cap \{i,j\}$. Suppose without loss of generality that $i\in S$ and $j\in T$. Since *S* is either a support or can be written as a union of nondisjoint supports that do not contain *j*, and *T* is either a support or can be written as a union of nondisjoint supports that do not contain *i*, the fact that $S \cup T$ can also be written as $(S \cup T) \cup \{i,j\}$, is a contradiction with ${{\mathcal {F}}}$ being cycle-free since $S\cup T$ can be written as union of non-unitary supports in more than one way. $\square$

When we restrict ourselves to the class of cycle-free accessible union stable network structures, we also need to verify if deleting all coalitions that contain two particular players, we still have a network structure that is, besides accessible and union stable, also cycle-free.

### Proposition 4.4

If ${{\mathcal {F}}}$ is a cycle-free accessible union stable network structure and $\{i,j\}\in B({{\mathcal {F}}})$ then ${{\mathcal {F}}}_{-ij}$ is a cycle-free accessible union stable network structure.

### Proof

From Propositions 4.2 (on accessibility) and 4.3 (on union stability) it follows that we only need to verify cycle-freeness (condition (ii) of Definition 3.1). Since every $S \in {{\mathcal {F}}}$ can be written as a union of non-unitary supports in a unique way, this obviously holds for every $S \in {{\mathcal {F}}} ^\prime \subseteq {{\mathcal {F}}}$, in particular for ${{\mathcal {F}}}^\prime = {{\mathcal {F}}} _{-ij}$. $\square$

Note that if ${{\mathcal {F}}}={{\mathcal {F}}}_L$ for some communication graph *L* then ${{\mathcal {F}}}_{-ij}$, in general, is not the set of connected coalitions in $L \setminus \{\{i,j\}\}$, as is illustrated by the accessible union stable network structure in Example 4.1 where $\{1,2,3,4\} \in {{\mathcal {F}}}_{L \setminus \{\{1,2\}\}} \setminus ({{\mathcal {F}}}_L)_{-12}$. Therefore, on the class of communication graph games, balanced fairness as defined in Definition 4.4 is not the same as Myerson’s fairness. However, for (sets of connected coalitions in) cycle-free graphs they are the same. Therefore, it is known that for these network structures, balanced fairness and component efficiency characterize the Shapley value. Similar, it follows that for the class games on cycle-free accessible union stable network structures, the Shapley value is characterized by component efficiency, component dummy and balanced fairness. We give an alternative proof considering this as a subclass of games on accessible union stable network structures and applying the results obtained for cycle-free accessible union stable network structures for completeness and to give a better understanding of these structures.

### Theorem 4.1

The Shapley value is the unique allocation rule on the class of games on a cycle-free accessible union stable network structure that satisfies component efficiency, component dummy and balanced fairness.

### Proof

The Shapley value satisfying component efficiency, component dummy and balanced fairness can be shown in a similar way as shown in Algaba et al. (2001). To show uniqueness, suppose that an allocation rule *f* satisfies component efficiency, component dummy and balanced fairness. We prove uniqueness (in a similar way to Myerson (1977) and Algaba et al. (2001)) by induction on the number of non-unitary coalitions in $${{\mathcal {F}}} $$. If $$|\{S\in {{\mathcal {F}}}\mid |S|\ge 2\}|=0$$ then every singleton is a component or a dummy, and then component efficiency or component dummy, respectively, determines the payoffs. Proceeding by induction, assume that $$ f(v,{{\mathcal {F}}})$$ is determined whenever $$\left| \{S\in {{\mathcal {F}}}\mid |S|\ge 2\}\right| <k$$, and suppose that $$\left| \{S\in {{\mathcal {F}}}\mid |S|\ge 2\}\right| =k$$. Observe that the grand coalition *N* is the union of the set of disjoint dummy players and the components of *N*. Therefore, it suffices to check that for every $$M\in C_{{{\mathcal {F}}}}\left( N\right) $$ it holds that $$f_{i}\left( v,{{\mathcal {F}}}\right) =\varphi _{i}\left( v,{{\mathcal {F}}}\right) $$ for all $$i\in M.$$ Let $$M\in C_{{{\mathcal {F}}}}(N)$$. If $$|M|=1$$ then component efficiency determines the payoff for $$i\in M$$. Otherwise, if $$ |M|>1$$, we know from Algaba et al. (2001) that we can label the players in $$M=\{i_{1},\ldots ,i_{m}\}$$ such that for every $$k\in \{1,\ldots , m-1\}$$ there is a support $$ H=\{i_{k},i_{k+1}\}$$. Since $${{\mathcal {F}}}_{-i_{k}i_{k+1}}$$ is a cycle-free accessible union stable network structure by Proposition 4.4, we can repeatedly apply balanced fairness yielding

4 $$\begin{aligned} f_{i_{k}}(v,{{\mathcal {F}}})\!-\!f_{i_{k}}(v,{{\mathcal {F}}}_{-i_{k}i_{k+1}})\!=\!f_{i_{k+1}}(v, {{\mathcal {F}}})-f_{i_{k+1}}(v,{{\mathcal {F}}}_{-i_{k}i_{k+1}}),\ k\!\in \! \{1,\ldots ,m-1\}.\nonumber \\ \end{aligned}$$

This yields $|M|-1$ linear independent equations. Component efficiency requires that

5 $$\begin{aligned} \sum _{i\in M}f_{i}\left( v,{{\mathcal {F}}}\right) =v(M). \end{aligned}$$

Since the values $f(v,{{\mathcal {F}}}_{-i_{k}i_{k+1}})$ are known by the induction hypothesis, together with the equations (4) and (5) this yields |*M*| linear independent equations in the |*M*| unknown payoffs $f_{h}(v,{{\mathcal {F}}})$, $h\in M$, which are therefore uniquely determined. $\square$

## Concluding remarks

One of the concluding remarks in Algaba et al. (2018) is that finding a suitable fairness axiom to characterize the Shapley value for games on accessible union stable network structures is a question for future research. A problem with applying balanced fairness as considered in this note is that union stability may be not preserved, and therefore the axiom might not have enough bite. This work partially answers this question in the sense that the balanced fairness axiom has enough strength in the special class of games on a cycle-free accessible union stable network structure.

**Fig. 1:**
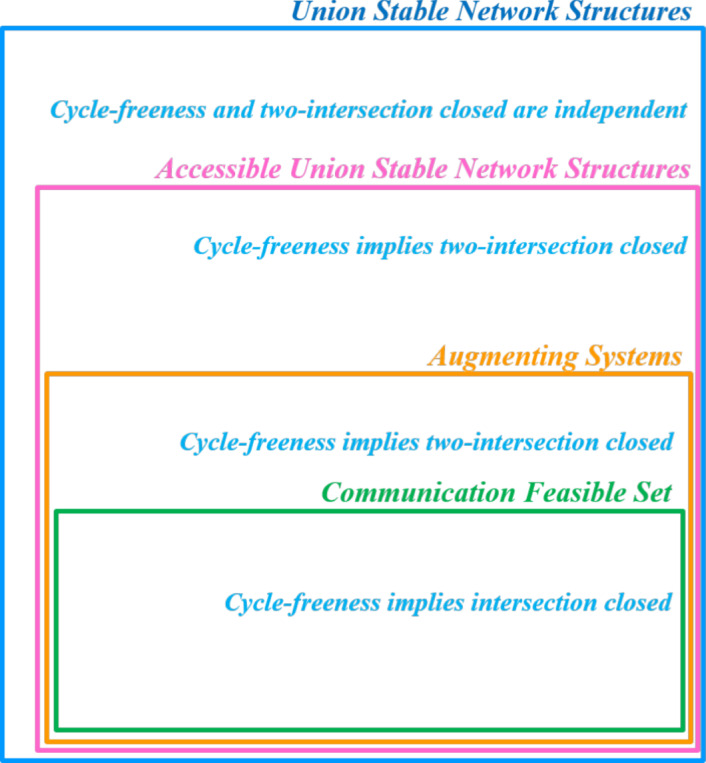
Venn diagram of network structures with respect to the inclusion.

In Figure 1, we summarize the logical relations between the various network structures mentioned in this paper. This paper focussed on the cycle-freeness property. Within the general class of union stable network structures cycle-freeness is not related to two-intersection closedness, but within the class of accessible union stable network structures, cycle-freeness implies two-intersection closedness.

Another question for future research is to study the relation between balanced fairness and the fairness axiom of Algaba et al. (2001) which generalizes Myerson (1977)’s fairness for communication graph games to union stable network structures. Notice that ${{\mathcal {B}}}({{\mathcal {F}}}) = {{\mathcal {B}}}(\overline{{\mathcal {F}}})$, with the union stable closure $\overline{{\mathcal {F}}}$ of any network structure ${{\mathcal {F}}} \subseteq 2^N$ given as in Footnote 10.

An allocation rule *f* on a subclass ${{\mathcal {C}}} \subseteq GUS^N$ of games on union stable network structures satisfies *fairness* on ${{\mathcal {C}}}$ if

$$\begin{aligned} f_i(v,{{\mathcal {F}}}) - f_i(v,\overline{{{\mathcal {B}}}({{\mathcal {F}}}) \setminus \{B\}}) = f_j(v,{{\mathcal {F}}}) - f_j(v,\overline{{{\mathcal {B}}}({{\mathcal {F}}}) \setminus \{B\}}), \end{aligned}$$

for all $\left( v,{{\mathcal {F}}}\right) \in {{\mathcal {C}}}$, $B \in {{\mathcal {B}}}({{\mathcal {F}}})$ and $i, j \in B$ such that $(v,\overline{{{\mathcal {B}}}({{\mathcal {F}}}) \setminus \{B\}}) \in {{\mathcal {C}}}$.

Looking at the communication graph described in Example 4.1, and observing that for that graph $\overline{{{\mathcal {B}}}({{\mathcal {F}}}) \setminus \{\{1,2\}\}}={{\mathcal {F}}}_{-12} \cup \{\{1,2,3,4\}\}$, it is clear that, for general communication graphs these axioms are different. However, it turns out that on the class of cycle-free accessible union stable network structures, these two axioms are equivalent.^17^ This follows from the following proposition.

### Proposition 5.1

For every cycle-free accessible union stable network structure ${{\mathcal {F}}}$ and support $\{i,j\} \in {{\mathcal {B}}}({{\mathcal {F}}}),\ i \ne j$, it holds that ${{\mathcal {F}}}_{-ij} = \overline{{{\mathcal {B}}}({{\mathcal {F}}}) \setminus \{\{i,j\}\}}$.

### Proof

$(\Rightarrow ): {{\mathcal {F}}}_{-ij} \subseteq \overline{{{\mathcal {B}}}({{\mathcal {F}}}) \setminus \{\{i,j\}\}}$. Let $S \in {{\mathcal {F}}}_{-ij}$. If $S \in {{\mathcal {B}}}({{\mathcal {F}}})$ then obviously $S \in {{\mathcal {B}}}({{\mathcal {F}}}) \setminus \{\{i,j\}\}$. If $S \not \in {{\mathcal {B}}}({{\mathcal {F}}})$ then *S* can be written as the union of two disjoint feasible coalitions that do not contain *i* and *j*, and thus $S \in \overline{{{\mathcal {B}}}({{\mathcal {F}}}) \setminus \{\{i,j\}\}}$.

$(\Leftarrow ): \overline{{{\mathcal {B}}}({{\mathcal {F}}}) \setminus \{\{i,j\}\}} \subseteq {{\mathcal {F}}}_{-ij}$. Let $S \in \overline{{{\mathcal {B}}}({{\mathcal {F}}}) \setminus \{\{i,j\}\}}$. If $\{i,j\} \not \subseteq S$ then $S \in {{\mathcal {F}}}_{-ij}$. Suppose by contradiction that $\{i,j\} \subseteq S$. Since $\{i,j\} \not \in \overline{{{\mathcal {B}}}({{\mathcal {F}}}) \setminus \{\{i,j\}\}}$, there exists a sequence of distinct coalitions $T_1, \ldots , T_m \in {{\mathcal {F}}}$ such that

(i) $$\bigcup _{k=1}^m T_k=S$$,

(ii) for every $k = 1, \ldots m-1$, $\left( \bigcup _{l=1}^k T_l \right) \cap T_{k+1} \ne \emptyset$, and

(iii) there exists an $r \in \{1, \ldots k\}$ such that $|\bigcup _{l=1}^r T_l \cap \{i,j\}|=|\bigcup _{l=r+1}^m T_l \cap \{i,j\}|=1$ with $\bigcup _{l=1}^r T_l \cap \{i,j\} \ne \bigcup _{l=r+1}^m T_l \cap \{i,j\}$.

Suppose without loss of generality that $\bigcup _{l=1}^r T_l \cap \{i,j\} = \{i\}$ and $\bigcup _{l=r+1}^m T_l \cap \{i,j\}=\{j\}$. Since $S \cup \{i,j\} = S$ can be written as the union of nondisjoint coalitions in more than one way (at least as $\left( \bigcup _{k=1}^{m-1} T_k\right) \cup T_m$ and $\left( \bigcup _{k=1}^m T_k\right) \cup \{i,j\}$), and therefore ${{\mathcal {F}}}$ is not cycle-free. $\square$

So, we have the following corollary on the class of cycle-free accessible union stable network structures.

### Corollary 5.1

An allocation rule *f* satisfies balanced fairness if and only if it satisfies fairness.

## Footnotes

1 This model is known under various names in the literature, such as communication situations, graph games, etc.

2 For cycle-free graph games, Ruyo et al. (2012) consider a parametrized version of fairness that also includes the component fairness axiom of Herings et al. (2008).

3 We remark that such structures are introduced in Algaba et al. (2000) under the name of union stable systems. Later, Faigle et al. (2010) established union stable systems as the more general systems where it is possible to define a meaningful notion of supermodularity that generalizes Shapley’s original convexity model for classical cooperative games. In their paper, union stable systems are renamed as weakly union-closed systems. Since, (i) union stable systems are also sometimes refering to the combination of a game with such a structure and (ii) this paper is about the special subclass of these structures introduced in Algaba et al. (2018) as accessible union stable networks structures, in this note we refer to them as union stable network structures.

4 In this context, Algaba et al. (2012) established connections between the Myerson and position values.

5 These solutions has been also studied in Algaba et al. (2020), for a particular case of union stable systems, allowing for analizing the feedback between the economic and political power of each coalition of agents.

6 As alternative to restricting the coalitions (i.e. sets of players) that are feasible, Faigle and Kern (1992) introduce games under precedence constraints where (i) the game is defined on a restricted domain that is determined by the hierarchy, and (ii) the possible orders in which coalitions can be formed is restricted. In this setting (Algaba et al. 2017) define a class of new values in which the removal of certain ‘irrelevant’ players does not effect the payoffs of the remaining players. A comparison between the two models is given in Algaba and van den Brink (2020).

7 An overview of games with communication and/or hierarchical properties can be found in Algaba and van den Brink (2021).

8 Balanced contributions is a ‘powerful’ axiom that also has been extended to axiomatize the Shapley value in other settings such as, for example, in multi-choice games in Hwang and Liao (2009).

9 Note that $C_L(S)$ is a partition of *S*.

10 Notice that this does not occur in a communication feasible set since all singletons are feasible.

11 For any network structure ${{\mathcal {F}}} \subseteq 2^N$ with $\emptyset \in {{\mathcal {F}}}$, let the *union stable closure* $\overline{{\mathcal {F}}}$ of ${{\mathcal {F}}}$ be given by the smallest (with respect to set inclusion) union stable network structure that contains ${{\mathcal {F}}}$.

12 Notice that, since this is a characterization within the class of union stable network structures, the empty set being feasible is implicitly assumed.

13 In Algaba et al. (2004) it is shown that the class of antimatroids that are intersection closed are exactly the poset antimatroids, that is, the antimatroids that can be the conjunctive feasible set of some acyclic permission structure.

14 In van den Brink (2012), Proposition 3.2 is stated using the *path property* instead of intersection closedness. This path property requires that for every pair of players $i, j \in N,\ i \ne j$, there is exactly one $\{i,j\}$-path, being a coalition *S* containing *i* and *j* such that *i* and *j* are the only nodes that can be removed from that coalition such that the remaining coalition is feasible (i.e. there exists $S \in {{\mathcal {F}}}$ with $i, j \in S$ such that $S\setminus \{i\} \in {{\mathcal {F}}},\ S \setminus \{j\} \in {{\mathcal {F}}}$ and $S \setminus \{h\} \not \in {{\mathcal {F}}}$ for all $h \in S \setminus \{i,j\}$). Under the assumptions of a communication feasible set, this is equivalent to intersection closedness.

15 These paths are used to characterize specific subclasses of antimatroids. For example, antimatroids ${{\mathcal {A}}}$ such that for every node $i \in N$ there is exactly one path $S \in {{\mathcal {A}}}$ such that $S \setminus \{i\} \in {{\mathcal {A}}}$, is the class of so-called poset antimatroids. This class is also characterized as the subclass of antimatroids that are closed under intersection, see Algaba et al. (2004).

16 So, we should call this axiom balanced fairness with respect to a certain class of games with restricted cooperation. When it does not lead to confusion we will simply refer to this axiom as balanced fairness.

17 We thank an anonymous reviewer for this suggestion.
